# Socioeconomic Inequalities in Oral Health Among Unmarried and Married Women: Evidence From a Population-Based Study in Japan

**DOI:** 10.2188/jea.JE20170088

**Published:** 2018-08-05

**Authors:** Keiko Murakami, Takayoshi Ohkubo, Hideki Hashimoto

**Affiliations:** 1 Department of Hygiene and Public Health, Teikyo University School of Medicine, Tokyo, Japan; 2 Department of Health and Social Behavior, School of Public Health, The University of Tokyo, Tokyo, Japan

**Keywords:** socioeconomic status, marital status, Japan, oral health, women

## Abstract

**Background:**

Socioeconomic inequalities in oral health have been reported in developed countries, but the influence of marital status has rarely been considered. Our aim was to examine marital status differentials in the association between socioeconomic status (SES) and oral health among community-dwelling Japanese women.

**Methods:**

From 2010 to 2011, a questionnaire survey was conducted among residents aged 25–50 years in Japanese metropolitan areas. Valid responses were received from 626 unmarried women and 1,620 married women. Women’s own and husbands’ educational attainment and equivalent income were used to assess SES. Self-rated “fair” or “poor” oral health was defined as poor oral health. Multiple logistic regression analysis was conducted to examine which SES indicators were associated with oral health.

**Results:**

The prevalence of poor oral health was 21.1% among unmarried women and 23.8% among married women. Among unmarried women, equivalent income was not associated with oral health, but women’s own education was significantly associated with oral health; the multivariate-adjusted odds ratio of poor oral health among those with high school education or lower compared to those with university education or higher was 2.14 (95% confidence interval, 1.19–3.87). Among married women, neither women’s own nor husbands’ education was associated with oral health, but equivalent income was significantly associated with oral health, particularly among housewives; the multivariate-adjusted odds ratio of poor oral health among those in the lowest compared with highest income quartile was 1.57 (95% confidence interval, 1.08–2.27).

**Conclusions:**

These findings indicate that marital status should be considered when examining associations between SES and oral health among Japanese women.

## INTRODUCTION

Many epidemiological studies have demonstrated an association between socioeconomic status (SES) and oral health in developed countries: lower SES groups have poorer oral health than higher ones.^1^^,^^2^ There is increasing evidence that socioeconomic inequalities in oral health exist also in Japan,^3^^–^^6^ where the public health insurance system has universally covered most dental care but not some forms of preventive dental care.^7^

We previously reported sex differences in associations between education and oral health in Japan: women’s education was not associated with oral health, but lower education was significantly associated with increased risk of poor oral health among men.^6^ However, assessment of socioeconomic inequalities in health among women poses a considerable challenge because of gender role norms, such as the male breadwinner model.^8^ The conventional sociological approach to conceptualizing married women’s SES assumes that women’s status is well represented by their husbands’ education and employment, not by their own.^9^ Even recent studies suggest that considering only women’s own SES may underestimate the magnitude of socioeconomic inequalities in health among women,^10^ despite the improvements in women’s economic independence arising from greater labor market participation. Although a few studies have demonstrated associations between husbands’ SES and women’s general health,^11^^–^^16^ to our knowledge, no studies have examined associations between husbands’ SES and women’s oral health.

As discussed above, the influence of SES on health may vary according to marital status; benefits from one’s own resources may be less important for married people, because they may enjoy some of these advantages through spousal supports.^13^^,^^17^ However, the possibility of marital status differentials is rarely taken into account, because most studies on socioeconomic inequalities in health have not separately analyzed data from unmarried and married individuals. Our previous study did not separately analyze data from unmarried and married women and did not consider the contribution of husbands’ education.^6^ Therefore, it is possible that the magnitude of socioeconomic inequalities in oral health among women has been underestimated.

Therefore, our aim was to examine differences in the association between SES, including husbands’ SES, and oral health according to marital status among community-dwelling Japanese women.

## METHODS

### Study design and participants

The present study was derived from the Japanese Study of Stratification, Health, Income, and Neighborhood (J-SHINE). This dataset has been described elsewhere.^6^^,^^18^^,^^19^ The J-SHINE survey was carried out in four municipalities in and around the greater Tokyo metropolitan area from October 2010 to February 2011. Of 13,920 adults aged 25–50 years who were probabilistically selected from the residential registry, survey staff members were able to contact 8,408 residents. Among them, 4,385 residents agreed to participate and complete the survey by providing their written consent (response rate: 31.5%; cooperation rate: 52.2%). After excluding those whose spouses wrongly answered, valid responses were received from 4,317 adults, 2,313 of whom were women. We analyzed 2,246 women (626 unmarried and 1,620 married) with no missing values on all variables used in the analysis other than income. The Research Ethics Committee of The University of Tokyo, Graduate School of Medicine approved the survey procedure of the J-SHINE (No. 3073). The J-SHINE Data Management Committee approved the authors’ secondary use of the data, with personally identifiable information deleted to ensure confidentiality.

### Measures

Marital status was measured using the question “Do you currently have a spouse/common-law partner?” Married participants were also asked when they got married. Participants reported their own educational attainment, and married participants also reported their spouses’ educational attainment. These were divided into three categories: high school or lower (elementary, junior high school, or senior high school), college (2-year college or special training school), and university or higher (university or graduate school). Participants selected their total annual household income from 15 response categories. Equivalent income was calculated as household income adjusted for household size, using the OECD-modified equivalence scale.^20^ This scale takes into account economies of scale in consumption and children’s lower consumption needs compared with those of adults by assigning lower weights to children than to adults. Because a substantial proportion of J-SHINE participants had children, this scale was adopted. For participants whose household income was unknown or missing but who responded on individual income, we used individual income as equivalent income. Income values that were missing after this step were imputed using a single imputation based on regression analysis that included other explanatory variables when logistic regression analyses were conducted. For married participants, spouses’ annual income was selected from 15 response categories, and values were assigned based on the midpoint of each category.

The main outcome was self-rated oral health, which is useful for assessing oral health in general populations.^21^^,^^22^ Self-rated oral health was measured using the question “How would you describe the health of your teeth and gums? Would you say it is excellent, very good, good, fair, or poor?” Responses were dichotomized as good oral health (excellent, very good, or good) and poor oral health (fair or poor) for analysis purposes.^6^ In the present study, we confirmed that poor self-rated oral health was significantly associated with the number of teeth removed (eTable 1).

As covariates, we chose age, municipality of residence, employment status (employed or unemployed), and psychological distress. Psychological distress was measured using the Japanese version of the K6 scale, which comprises six items assessing depressive moods and anxiety over the preceding 4 weeks on a five-point scale ranging from 0 (none of the time) to 4 (all of the time) (total score range, 0–24).^23^^,^^24^ We used a cut-off score of 5 to identify cases with psychological distress.^24^^,^^25^

### Statistical analysis

Characteristics of unmarried and married women were compared using the chi-squared test for categorical variables and Student’s *t* test for continuous variables. Multiple logistic regression analyses were conducted to examine the association of education and income with oral health. First, we examined whether marital status modified these associations by including the interaction term in the models. The results found that the interaction between marital status and women’s education was statistically significant (*P* = 0.032) but that there was no significant interaction between marital status and equivalent income (*P* = 0.48). Based on these results, we conducted analyses separately for unmarried and married women. For unmarried women, the odds ratios (ORs) and 95% confidence intervals (CIs) were calculated for women’s own education or equivalent income adjusted for age and municipality of residence (model 1), as well as for employment status and psychological distress (model 2). In model 3, we simultaneously entered education and income into the model to examine the independent association of each SES indicator. For married women, these analyses were conducted with husbands’ education as an added explanatory variable.

Previous research shows that socioeconomic inequalities in general health manifest in different ways according to age,^26^ but it is unclear whether such age-related differences also occur for oral health. Therefore, we also conducted stratified analyses by age category (divided by median age according to marital status) and examined whether age modified the associations between SES and oral health by including interaction terms in the models. Stratified analyses were also conducted by married women’s employment status to test whether the association between income and oral health differed according to women’s dependence on husbands’ earnings. Additionally, we calculated the OR and 95% CI for husbands’ income as an explanatory variable.

All analyses were conducted using Stata 12.0 (StataCorp LP, College Station, TX, USA), and a two-tailed *P* < 0.05 was considered statistically significant.

## RESULTS

The characteristics of respondents by marital status are shown in Table 1. Married women were older, were less educated, had higher equivalent incomes, were less likely to be employed, and had lower prevalence of psychological distress than were unmarried women. The percentages of poor oral health were 21.1% and 23.8% among unmarried and married women, respectively.

**Table 1.** Characteristics of unmarried women and married women

	Unmarried (*n* = 626)	Married (*n* = 1,620)	*P* value^a^
Age, years, mean (SD)	33.7 (7.1)	38.6 (6.8)	<0.001
Women’s educational attainment, *n* (%)			<0.001
University or higher	282 (45.0)	468 (28.9)	
College	236 (37.7)	752 (46.4)	
High school or lower	108 (17.3)	400 (24.7)	
Husbands’ educational attainment, *n* (%)			
University or higher		957 (59.1)	
College		299 (18.4)	
High school or lower		364 (22.5)	
Equivalent income, thousand JPY/year, mean (SD)	3141.8 (2122.3)	3463.7 (1996.1)	0.001
Employed, *n* (%)	560 (89.5)	929 (57.4)	<0.001
Psychological distress, *n* (%)	265 (42.3)	491 (30.3)	<0.001
Poor oral health, *n* (%)	132 (21.1)	386 (23.8)	0.167
Current smoker,^b^ *n* (%)	93 (14.9)	210 (13.0)	0.245
Preventive dental care use,^b^ *n* (%)	187 (30.1)	539 (33.4)	0.132
Duration of marriage (years),^b^ mean (SD)		11.7 (7.4)	

JPY, Japanese yen; SD, standard deviation.

^a^Obtained using the chi-squared test for categorical variables and Student’s *t* test for continuous variables, comparing unmarried and married women.

^b^Data on smoking status, preventive dental care use, and duration of marriage were available for 2,243, 2,236, and 1,506 participants, respectively.

Table 2 shows associations of education and income with oral health among unmarried women. A lower level of women’s own education was significantly associated with increased risk of poor oral health among unmarried women after adjusting for age, municipality, employment status, and psychological distress (model 2); the multivariate-adjusted OR of high school education or lower compared with university education or higher was 2.35 (95% CI, 1.34–4.12). The association did not materially change after further adjustment for equivalent income; the corresponding OR was 2.14 (95% CI, 1.19–3.87) (model 3). Equivalent income was not significantly associated with oral health. No significant interaction was found between age and SES variables (eTable 2).

**Table 2.** Prevalence and odds ratios for poor oral health among unmarried women (*n* = 626)

	%	Model 1 OR (95% CI)	Model 2 OR (95% CI)	Model 3 OR (95% CI)
Women’s educational attainment				
University or higher	20.7	1.00	1.00	1.00
College	21.7	1.59 (1.00–2.54)	1.51 (0.94–2.43)	1.42 (0.87–2.32)
High school or lower	29.3	2.51 (1.44–4.37)	2.35 (1.34–4.12)	2.14 (1.19–3.87)
Equivalent income				
4th quartile	16.1	1.00	1.00	1.00
3rd quartile	19.5	1.34 (0.74–2.43)	1.31 (0.72–2.40)	1.17 (0.64–2.17)
2nd quartile	21.5	1.54 (0.86–2.75)	1.42 (0.79–2.56)	1.17 (0.63–2.17)
1st quartile (lowest)	27.0	2.00 (1.14–3.49)	1.74 (0.98–3.09)	1.39 (0.75–2.54)

CI, confidence interval; OR, odds ratio.

Model 1: adjusted for age and municipality.

Model 2: model 1 + adjusted for employment status and psychological distress.

Model 3: model 2 + adjusted for women’s educational attainment/equivalent income.

Table 3 shows associations of education and income with oral health among married women. Women’s own educational attainment was not significantly associated with oral health. Husbands’ educational attainment was significantly associated with oral health after adjusting for age, municipality, employment status, and psychological distress (model 2), but this association became non-significant after further adjustment for women’s own education and equivalent income (model 3). Lower equivalent income was significantly associated with increased risk of poor oral health even after adjusting for age, municipality, employment status, and psychological distress (model 2); the multivariate-adjusted OR of the lowest income quartile compared with the highest income quartile was 1.67 (95% CI, 1.18–2.38). The association did not materially change after further adjustment for women’s and husbands’ educational attainment; the corresponding OR was 1.57 (95% CI, 1.08–2.27) (model 3). No significant interaction was found between age and SES variables (eTable 3).

**Table 3.** Prevalence and odds ratios for poor oral health among married women (*n* = 1,620)

	%	Model 1 OR (95% CI)	Model 2 OR (95% CI)	Model 3 OR (95% CI)
Women’s educational attainment				
University or higher	23.7	1.00	1.00	1.00
College	21.3	0.86 (0.65–1.14)	0.86 (0.65–1.14)	0.76 (0.56–1.02)
High school or lower	28.8	1.25 (0.91–1.70)	1.26 (0.92–1.72)	1.01 (0.71–1.43)
Husbands’ educational attainment				
University or higher	21.7	1.00	1.00	1.00
College	24.8	1.21 (0.89–1.65)	1.21 (0.89–1.65)	1.14 (0.83–1.58)
High school or lower	28.6	1.39 (1.05–1.83)	1.39 (1.05–1.84)	1.26 (0.92–1.72)
Equivalent income				
4th quartile	18.7	1.00	1.00	1.00
3rd quartile	23.8	1.51 (1.06–2.14)	1.48 (1.04–2.10)	1.46 (1.02–2.09)
2nd quartile	25.7	1.63 (1.16–2.30)	1.60 (1.13–2.26)	1.52 (1.05–2.18)
1st quartile (lowest)	26.9	1.76 (1.24–2.49)	1.67 (1.18–2.38)	1.57 (1.08–2.27)

CI, confidence interval; OR, odds ratio.

Model 1: adjusted for age and municipality.

Model 2: model 1 + adjusted for employment status and psychological distress.

Model 3: model 2 + adjusted for women’s educational attainment/husbands’ educational attainment/equivalent income.

Table 4 shows the results of stratified analyses by married women’s employment status. Women’s own educational attainment was not associated with oral health irrespective of employment status, whereas low educational attainment in husbands was significantly associated with increased risk of poor oral health among married working women. Lower equivalent income was significantly associated with increased risk of poor oral health only among housewives. No significant interactions were detected between employment status and SES (assessed via women’s own educational attainment, husbands’ educational attainment, or equivalent income) (all *P* > 0.08). Lower husbands’ income was significantly associated with increased risk of poor oral health among housewives but not among married working women, although no significant interaction was found between employment status and husbands’ income (Table 5).

**Table 4.** Prevalence and odds ratios for poor oral health by employment status among married women (*n* = 1,620)

	%	Model 1 OR (95% CI)	Model 2 OR (95% CI)	Model 3 OR (95% CI)
Unemployed (*n* = 691)				
Women’s educational attainment				
University or higher	24.7	1.00	1.00	1.00
College	22.8	0.89 (0.58–1.36)	0.89 (0.58–1.36)	0.81 (0.52–1.26)
High school or lower	27.0	1.12 (0.69–1.83)	1.12 (0.68–1.83)	0.94 (0.55–1.61)
Husbands’ educational attainment				
University or higher	22.9	1.00	1.00	1.00
College	26.1	1.16 (0.72–1.86)	1.10 (0.68–1.78)	1.00 (0.61–1.64)
High school or lower	26.9	1.17 (0.76–1.81)	1.15 (0.74–1.78)	1.01 (0.62–1.64)
Equivalent income				
4th quartile	13.2	1.00	1.00	1.00
3rd quartile	25.6	2.31 (1.26–4.25)	2.33 (1.26–4.29)	2.34 (1.26–4.33)
2nd quartile	28.1	2.58 (1.38–4.81)	2.59 (1.38–4.85)	2.65 (1.38–5.07)
1st quartile (lowest)	27.1	2.43 (1.30–4.54)	2.29 (1.22–4.29)	2.34 (1.21–4.52)
Employed (*n* = 929)				
Women’s educational attainment				
University or higher	23.0	1.00	1.00	1.00
College	20.1	0.81 (0.56–1.18)	0.81 (0.56–1.19)	0.72 (0.48–1.07)
High school or lower	30.0	1.34 (0.89–2.02)	1.37 (0.91–2.07)	1.14 (0.72–1.82)
Husbands’ educational attainment				
University or higher	20.8	1.00	1.00	1.00
College	23.9	1.25 (0.83–1.88)	1.26 (0.84–1.90)	1.28 (0.83–1.97)
High school or lower	29.8	1.55 (1.07–2.24)	1.56 (1.08–2.27)	1.52 (1.00–2.30)
Equivalent income				
4th quartile	21.2	1.00	1.00	1.00
3rd quartile	26.8	1.54 (0.98–2.42)	1.53 (0.98–2.41)	1.51 (0.95–2.40)
2nd quartile	19.5	1.03 (0.65–1.63)	1.01 (0.64–1.60)	0.90 (0.55–1.46)
1st quartile (lowest)	26.9	1.51 (0.97–2.34)	1.46 (0.93–2.27)	1.28 (0.80–2.06)

CI, confidence interval; OR, odds ratio.

Interaction between employment status and women’s educational attainment: model 1, *P* = 0.435; model 2, *P* = 0.383; model 3, *P* = 0.456.

Interaction between employment status and husbands’ educational attainment: model 1, *P* = 0.420; model 2, *P* = 0.346; model 3, *P* = 0.348.

Interaction between employment status and equivalent income: model 1, *P* = 0.086; model 2, *P* = 0.099; model 3, *P* = 0.114.

Model 1: adjusted for age and municipality.

Model 2: model 1 + adjusted for psychological distress.

Model 3: model 2 + adjusted for women’s educational attainment/husbands’ educational attainment/equivalent income.

**Table 5.** Prevalence and odds ratios for poor oral health according to husbands’ income among married women

	%	Model 1 OR (95% CI)	Model 2 OR (95% CI)	Model 3 OR (95% CI)
Total (*n* = 1,303)				
Husbands’ income				
4th quartile	19.6	1.00	1.00	1.00
3rd quartile	21.1	1.10 (0.65–1.87)	1.10 (0.64–1.87)	1.06 (0.62–1.82)
2nd quartile	23.9	1.39 (0.86–2.25)	1.40 (0.87–2.27)	1.33 (0.81–2.18)
1st quartile (lowest)	27.3	1.72 (1.03–2.86)	1.62 (0.96–2.72)	1.49 (0.87–2.57)
Unemployed (*n* = 570)				
Husbands’ income				
4th quartile	15.1	1.00	1.00	1.00
3rd quartile	16.5	1.12 (0.49–2.55)	1.10 (0.48–2.53)	1.08 (0.47–2.50)
2nd quartile	24.3	1.86 (0.88–3.96)	1.84 (0.86–3.92)	1.81 (0.83–3.93)
1st quartile (lowest)	29.7	2.51 (1.18–5.37)	2.35 (1.10–5.05)	2.32 (1.04–5.16)
Employed (*n* = 731)				
Husbands’ income				
4th quartile	24.6	1.00	1.00	1.00
3rd quartile	25.2	1.02 (0.50–2.07)	1.01 (0.49–2.05)	0.97 (0.47–1.99)
2nd quartile	20.3	0.87 (0.46–1.67)	0.86 (0.45–1.65)	0.83 (0.43–1.62)
1st quartile (lowest)	28.8	1.54 (0.78–3.02)	1.43 (0.73–2.83)	1.34 (0.66–2.73)

CI, confidence interval; OR, odds ratio.

Interaction between employment status and husbands’ income: model 1, *P* = 0.180; model 2, *P* = 0.197; model 3, *P* = 0.212.

Model 1: adjusted for age and municipality.

Model 2: model 1 + adjusted for psychological distress.

Model 3: model 2 + adjusted for women’s educational attainment and husbands’ educational attainment.

## DISCUSSION

The present study explored socioeconomic inequalities in oral health among unmarried and married women in Japan. A lower level of women’s own education was significantly associated with increased risk of poor oral health only among unmarried women. Among married women, neither women’s own nor husbands’ education was associated with oral health, but lower equivalent income was significantly associated with increased risk of poor oral health, particularly among housewives.

Distinct associations between SES and oral health by marital status were observed: women’s own education was associated with oral health among unmarried women, whereas income was associated with oral health among married women. Although different SES indicators reflect core dimensions of social stratification, they have different societal meanings.^26^^,^^27^ Women are more susceptible than men to social influence and to others’ attitudes toward their behaviors.^28^^,^^29^ Marriage may bring about a drastic change in the sources of social influence on women’s health.

An inverse association between education and oral health was observed among unmarried women, but not among married women, with significant interaction by marital status. Education may influence health through the knowledge and skills attained; these may have an influence on people’s cognitive function and make them more receptive to messages about health education.^26^^,^^30^ However, it is highly unlikely that marriage decreases the effect of the knowledge and skills attained through education, which suggests that this effect cannot fully explain education-related inequalities in oral health.

Education and lifelong socialization shape cultural capital in the form of health values and behavioral norms,^31^ and cultural values related to oral health influence the adoption of efficacious preventive behaviors.^32^ As a form of cultural values and norms, education may affect how much an individual is influenced by societal standards underlying health-related messages (including those about oral health).^33^^,^^34^ The association between education and health behaviors can also be explained by social networks,^30^ which combine individual resources, such as education, with those of others.^35^ As associations between social networks and oral health have been demonstrated,^36^ the influence of social networks on associations of education with health behaviors could be applied to oral health practices. We previously showed in the J-SHINE survey^18^ that a higher level of education was significantly associated with preventive dental care use among women; those results may partially explain the behavioral pathway through which education is associated with oral health.

Among married women, lower equivalent income was significantly associated with increased risk of poor oral health, particularly among housewives. Marriage generally has a beneficial effect on health,^37^ mainly owing to financial resources, among women.^38^^,^^39^ Particularly in Japanese society, gender role norms (ie, the male breadwinner model) are relatively strong.^8^ We also found that husbands’ income was significantly associated with oral health among housewives but not among married working women. Income reflects social standing as well as accessibility to material conditions that affect health.^26^ For housewives, therefore, financial situation (particularly husbands’ earnings) is an important indicator of their position in society.

Husbands’ education was significantly associated with oral health after adjusting for age, municipality, employment status, and psychological distress, but this association became non-significant after further adjustment for women’s own education and equivalent income. This result is inconsistent with several studies that have demonstrated inverse associations between husbands’ education and self-rated health^11^^,^^14^ or mortality.^12^^,^^13^^,^^15^^,^^16^ Although oral health and general health (eg, non-communicable chronic diseases) share common risk factors,^40^ some aspects of oral health differ from those of general health; for example, the occurrence of oral diseases is more predictable, and there is probably a wider variety of alternative treatments available for oral diseases.^41^ These differences may explain why oral health and general health measures do not show similar associations with other factors. Another explanation for this inconsistent result may be that any effect of husbands’ education on oral health is relatively late. Oral health is cumulative and best considered from a life-course perspective. Research indicates that adult oral health is influenced by childhood social conditions.^42^ We previously showed in the J-SHINE survey^6^ that childhood economic status, but not parental education, was significantly associated with oral health among women. The nonsignificant association between husbands’ education and oral health could also be a result of over-adjustment for SES indicators. Although each SES indicator represents a distinct aspect of a person’s social position,^26^^,^^27^ these indicators are correlated. Simultaneous adjustment for these SES indicators may have resulted in over-adjustment.

These findings have several implications for health policy. Marital status differences in the associations of education and income with oral health suggest that public health interventions should consider the social context in which women live. Combined analyses of data from unmarried and married women cannot provide a complete picture of socioeconomic inequalities in health. Dental care offers extensive prevention possibilities that can save resources, which is often not the case in other forms of medical care.^41^ Therefore, it would be beneficial to examine socioeconomic inequalities in oral health in more detail.

Limitations of the present study should be noted. First, the J-SHINE survey had a relatively low response rate, although the respondents were fairly comparable with the target population in age, sex, and educational attainment.^19^ Second, oral health status was self-reported. Self-rated oral health is used frequently in population-based studies when clinical evaluations are too costly and is a valid and useful summary indicator of overall oral health status.^21^^,^^22^ Although poor self-rated oral health as defined in the present study was significantly associated with the number of teeth removed (eTable 1), the J-SHINE survey did not measure periodontal conditions or oral health-related quality of life. It would be interesting to examine associations between these other types of oral health measures and SES. Third, we had no detailed information about unmarried status and could not differentiate between single, divorced, and widowed unmarried status. As unmarried women in the present study were relatively young, probably very few were widows. Finally, because this was a cross-sectional study, the causal direction of the observed associations could not be determined. Although it is unlikely that adult oral health affects education, it may have some influence on income and husbands’ education.

In conclusion, the present study found that a lower level of women’s own education was significantly associated with increased risk of poor oral health only among unmarried women. Among married women, neither women’s own nor husbands’ education was associated with oral health, but lower income was significantly associated with increased risk of poor oral health, particularly among housewives. These findings indicate that marital status should be considered when examining associations between SES and oral health among Japanese women and could be useful for public health intervention initiatives to reduce socioeconomic inequalities in oral health.

## APPENDIX A. SUPPLEMENTARY DATA

eTable l. Prevalence of poor oral health according to number of teeth removed

eTable 2. Odds ratios for poor oral health among unmarried women by age category

eTable 3. Odds ratios for poor oral health among married women by age category

## References

[1] Watt RG From victim blaming to upstream action: tackling the social determinants of oral health inequalities. Community Dent Oral Epidemiol. 2007;35:1–11. 10.1111/j.1600-0528.2007.00348.x 17244132

[2] Petersen PE, Kwan S Equity, social determinants and public health programmes—the case of oral health. Community Dent Oral Epidemiol. 2011;39:481–487. 10.1111/j.1600-0528.2011.00623.x 21623864

[3] Ueno M, Ohara S, Inoue M, Tsugane S, Kawaguchi Y Association between education level and dentition status in Japanese adults: Japan public health center-based oral health study. Community Dent Oral Epidemiol. 2012;40:481–487. 10.1111/j.1600-0528.2012.00697.x 22537553

[4] Ando A, Ohsawa M, Yaegashi Y, Factors related to tooth loss among community-dwelling middle-aged and elderly Japanese men. J Epidemiol. 2013;23:301–306. 10.2188/jea.JE20120180 23812101 PMC3709550

[5] Ito K, Aida J, Yamamoto T, ; JAGES Group Individual- and community-level social gradients of edentulousness. BMC Oral Health. 2015;15:34. 10.1186/s12903-015-0020-z 25884467 PMC4460930

[6] Murakami K, Kondo N, Ohkubo T, Hashimoto H The effect of fathers’ and mothers’ educational level on adult oral health in Japan. Community Dent Oral Epidemiol. 2016;44:283–291. 26871456 10.1111/cdoe.12216

[7] Ikegami N, Yoo BK, Hashimoto H, Japanese universal health coverage: evolution, achievements, and challenges. Lancet. 2011;378:1106–1115. 10.1016/S0140-6736(11)60828-3 21885107

[8] Brinton M. *Women and the economic miracle. Gender and work in postwar Japan*. Berkeley and Los Angeles, CA: University of California Press; 1993.

[9] Shirahase S Women and class structure in contemporary Japan. Br J Sociol. 2001;52:391–408. 10.1080/00071310120071115 11578002

[10] Honjo K, Iso H, Iwata M, ; JPHC Study Group Effectiveness of the combined approach for assessing social gradients in stroke risk among married women in Japan. J Epidemiol. 2012;22:324–330. 10.2188/jea.JE20110147 22522151 PMC3798651

[11] Monden CW, van Lenthe F, de Graaf ND, Kraaykamp G Partner’s and own education: does who you live with matter for self-assessed health, smoking and excessive alcohol consumption? Soc Sci Med. 2003;57:1901–1912. 10.1016/S0277-9536(03)00055-8 14499514

[12] Jaffe DH, Eisenbach Z, Neumark YD, Manor O Effects of husbands’ and wives’ education on each other’s mortality. Soc Sci Med. 2006;62:2014–2023. 10.1016/j.socscimed.2005.08.030 16199120

[13] Kravdal Ø A broader perspective on education and mortality: are we influenced by other people’s education? Soc Sci Med. 2008;66:620–636. 10.1016/j.socscimed.2007.10.009 18023954

[14] Brown DC, Hummer RA, Hayward MD The importance of spousal education for the self-rated health of married adults in the United States. Popul Res Policy Rev. 2014;33:127–151. 10.1007/s11113-013-9305-6 24511172 PMC3912877

[15] Spoerri A, Schmidlin K, Richter M, Egger M, Clough-Gorr KM; Swiss National Cohort (SNC) Individual and spousal education, mortality and life expectancy in Switzerland: a national cohort study. J Epidemiol Community Health. 2014;68:804–810. 10.1136/jech-2013-203714 24764353

[16] Yang L, Martikainen P, Silventoinen K Effects of individual, spousal, and offspring socioeconomic status on mortality among elderly people in China. J Epidemiol. 2016;26:602–609. 10.2188/jea.JE20150252 27150012 PMC5083324

[17] Smith KR, Zick CD Linked lives, dependent demise? Survival analysis of husbands and wives. Demography. 1994;31:81–93. 10.2307/2061909 8005344

[18] Murakami K, Aida J, Ohkubo T, Hashimoto H Income-related inequalities in preventive and curative dental care use among working-age Japanese adults in urban areas: a cross-sectional study. BMC Oral Health. 2014;14:117. 10.1186/1472-6831-14-117 25234486 PMC4176863

[19] Takada M, Kondo N, Hashimoto H; J-SHINE Data Management Committee Japanese study on stratification, health, income, and neighborhood: study protocol and profiles of participants. J Epidemiol. 2014;24:334–344. 10.2188/jea.JE20130084 24814507 PMC4074639

[20] Hagenaars AJM, de Vos K, Zaidi MA. *Poverty Statistics in the late 1980s: research based on micro-data*. Luxembourg: Office for Official Publications of the European Communities; 1994.

[21] Locker D, Miller Y Evaluation of subjective oral health status indicators. J Public Health Dent. 1994;54:167–176. 10.1111/j.1752-7325.1994.tb01209.x 7932353

[22] Jones JA, Kressin NR, Miller DR, Orner MB, Garcia RI, Spiro A 3rd Comparison of patient-based oral health outcome measures. Qual Life Res. 2004;13:975–985. 10.1023/B:QURE.0000025596.05281.d6 15233511

[23] Kessler RC, Andrews G, Colpe LJ, Short screening scales to monitor population prevalences and trends in non-specific psychological distress. Psychol Med. 2002;32:959–976. 10.1017/S0033291702006074 12214795

[24] Furukawa TA, Kawakami N, Saitoh M, The performance of the Japanese version of the K6 and K10 in the World Mental Health Survey Japan. Int J Methods Psychiatr Res. 2008;17:152–158. 10.1002/mpr.257 18763695 PMC6878390

[25] Sakurai K, Nishi A, Kondo K, Yanagida K, Kawakami N Screening performance of K6/K10 and other screening instruments for mood and anxiety disorders in Japan. Psychiatry Clin Neurosci. 2011;65:434–441. 10.1111/j.1440-1819.2011.02236.x 21851452

[26] Galobardes B, Shaw M, Lawlor DA, Davey Smith G, Lynch JW. Indicators of socioeconomic position. In: Oakes JM, Kaufman JS, eds. *Methods in social epidemiology*. San Francisco, CA: Jossey-Bass; 2006:47–85.

[27] Murakami K, Hashimoto H, Lee JS, Kawakubo K, Mori K, Akabayashi A Distinct impact of education and income on habitual exercise: a cross-sectional analysis in a rural city in Japan. Soc Sci Med. 2011;73:1683–1688. 10.1016/j.socscimed.2011.09.024 22033375

[28] Cross SE, Madson L Models of the self: self-construals and gender. Psychol Bull. 1997;122:5–37. 10.1037/0033-2909.122.1.5 9204777

[29] Chao D, Hashimoto H, Kondo N Dynamic impact of social stratification and social influence on smoking prevalence by gender: an agent-based model. Soc Sci Med. 2015;147:280–287. 10.1016/j.socscimed.2015.08.041 26610078

[30] Cutler DM, Lleras-Muney A Understanding differences in health behaviors by education. J Health Econ. 2010;29:1–28. 10.1016/j.jhealeco.2009.10.003 19963292 PMC2824018

[31] Lareau A, Weininger EB Cultural capital in educational research: a critical assessment. Theory Soc. 2003;32:567–606. 10.1023/B:RYSO.0000004951.04408.b0

[32] Patrick DL, Lee RS, Nucci M, Grembowski D, Jolles CZ, Milgrom P Reducing oral health disparities: a focus on social and cultural determinants. BMC Oral Health. 2006;6(Suppl 1):S4. 10.1186/1472-6831-6-S1-S4 16934121 PMC2147600

[33] Bourdieu P. *Distinction: a social critique of the judgement of taste*. London: Routledge; 1984.

[34] Abel T Cultural capital and social inequality in health. J Epidemiol Community Health. 2008;62:e13. 10.1136/jech.2007.066159 18572429

[35] Berkman LF, Krishna A. Social network epidemiology. In: Berkman LF, Kawachi I, Glymour MM, eds. *Social epidemiology*. New York, NY: Oxford University Press; 2014:234–289.

[36] Rouxel PL, Heilmann A, Aida J, Tsakos G, Watt RG Social capital: theory, evidence, and implications for oral health. Community Dent Oral Epidemiol. 2015;43:97–105. 10.1111/cdoe.12141 25533022

[37] Manzoli L, Villari P, M Pirone G, Boccia A Marital status and mortality in the elderly: a systematic review and meta-analysis. Soc Sci Med. 2007;64:77–94. 10.1016/j.socscimed.2006.08.031 17011690

[38] Lillard LA, Waite LJ Til death do us part: marital disruption and mortality. Am J Sociol. 1995;100:1131–1156. 10.1086/230634

[39] Maselko J, Bates LM, Avendaño M, Glymour MM The intersection of sex, marital status, and cardiovascular risk factors in shaping stroke incidence: results from the health and retirement study. J Am Geriatr Soc. 2009;57:2293–2299. 10.1111/j.1532-5415.2009.02555.x 19874408

[40] Watt RG, Sheiham A Integrating the common risk factor approach into a social determinants framework. Community Dent Oral Epidemiol. 2012;40:289–296. 10.1111/j.1600-0528.2012.00680.x 22429083

[41] Sintonen H, Linnosmaa I. Economics of dental services. In: Culyer AJ, Newhouse JP, eds. *Handbook of Health Economics, volume 1B*. Amsterdam: Elsevier; 2000:1251–1296.

[42] Nicolau B, Thomson WM, Steele JG, Allison PJ Life-course epidemiology: concepts and theoretical models and its relevance to chronic oral conditions. Community Dent Oral Epidemiol. 2007;35:241–249. 10.1111/j.1600-0528.2007.00332.x 17615010

