# Supplementary material for: Socioeconomic Inequalities in Oral Health Among Unmarried and Married Women: Evidence From a Population-Based Study in Japan
Source: J Epidemiol. 2018 Aug 5;28(8):341–6. doi: 10.2188/jea.JE20170088 (PMC6048301; doi:10.2188/jea.JE20170088)
Supplement: Supplementary file 1 [file je-28-341-s001.pdf]

eTable 1. Prevalence of poor oral health according to number of teeth removed

Number of teeth removed	Number with poor oral health / Participants	(%)	*P* value[a]
0	209 / 1,307	(16.0)	<0.001
1	76 / 295	(25.8)	
2	46 / 163	(28.2)	
3	32 / 87	(36.8)	
4	30 / 71	(42.3)	
≥5	59 / 116	(50.9)	

[a] Obtained using the chi-squared test.

eTable 2. Odds ratios for poor oral health among unmarried women by age category

	25–32 years (n=319)<br>OR (95% CI)	33–50 years (n=307)<br>OR (95% CI)	*P* for interaction[a]
Women's educational attainment			0.476
University or higher	1.00	1.00	
College	1.47 (0.73–2.97)	1.26 (0.61–2.59)	
High school or lower	2.42 (0.99–5.92)	1.68 (0.72–3.93)	
Equivalent income			0.741
4th quartile	1.00	1.00	
3rd quartile	0.66 (0.26–1.67)	1.91 (0.81–4.48)	
2nd quartile	1.04 (0.42–2.56)	1.29 (0.51–3.29)	
1st quartile (lowest)	1.32 (0.53–3.28)	1.65 (0.69–3.93)	

CI, confidence interval; OR, odds ratio.
Adjusted for age, municipality, employment status, psychological distress, and women's educational attainment/equivalent income.
[a] *P* denotes the significance of interaction between age categories (25–32 years and 33–50 years) and each variable.

eTable 3. Odds ratios for poor oral health among married women by age category

	25–39 years (n=848)<br>OR (95% CI)	40–50 years (n=772)<br>OR (95% CI)	*P* for interaction[a]
Women's educational attainment			0.333
University or higher	1.00	1.00	
College	0.61 (0.41–0.92)	0.96 (0.62–1.51)	
High school or lower	0.84 (0.52–1.35)	1.38 (0.82–2.33)	
Husbands' educational attainment			0.445
University or higher	1.00	1.00	
College	1.25 (0.81–1.92)	1.18 (0.71–1.96)	
High school or lower	1.44 (0.93–2.23)	1.16 (0.74–1.82)	
Equivalent income			0.104
4th quartile	1.00	1.00	
3rd quartile	1.42 (0.84–2.40)	1.60 (0.97–2.64)	
2nd quartile	1.39 (0.78–2.49)	1.29 (0.76–2.19)	
1st quartile (lowest)	2.00 (1.14–3.49)	1.21 (0.71–2.06)	

CI, confidence interval; OR, odds ratio.
Adjusted for age, municipality, employment status, psychological distress, and women's educational attainment/husbands' educational attainment/equivalent income.
[a] *P* denotes the significance of interaction between age categories (25–39 years and 40–50 years) and each variable.
